# Effect of *Moringa oleifera* Leaf Powder on Postprandial Blood Glucose Response: In Vivo Study on Saharawi People Living in Refugee Camps

**DOI:** 10.3390/nu10101494

**Published:** 2018-10-12

**Authors:** Alessandro Leone, Simona Bertoli, Sara Di Lello, Angela Bassoli, Stefano Ravasenghi, Gigliola Borgonovo, Fabio Forlani, Alberto Battezzati

**Affiliations:** 1 International Center for the Assessment of Nutritional Status (ICANS), University of Milan, Via Sandro Botticelli 21, 20133 Milan, Italy; simona.bertoli@unimi.it (S.B.); stefano.ravasenghi@unimi.it (S.R.); alberto.battezzati@unimi.it (A.B.); 2 Department of Food, Environmental and Nutritional Sciences (DeFENS), University of Milan, Via Luigi Mangiagalli 25, 20133 Milan, Italy; angela.bassoli@unimi.it (A.B.); gigliola.borgonovo@unimi.it (G.B.); fabio.forlani@unimi.it (F.F.); 3 Movimento Africa 70 NGO, Via Missori 14, 20900 Monza, Italy; sara.dilello71@gmail.com

**Keywords:** *Moringa oleifera*, diabetes, sensory acceptability, nutritional composition, humans

## Abstract

The hypoglycemic effect in humans of *Moringa oleifera* (MO) leaf powder has, to date, been poorly investigated. We assessed the chemical composition of MO leaf powder produced at Saharawi refugee camps, its in vitro ability to inhibit α-amylase activity, and its sensory acceptability in food. We then evaluated its effect on postprandial glucose response by randomly administering, on 2 different days, a traditional meal supplemented with 20 g of MO leaf powder (MOR20), or not (control meal, CNT), to 17 Saharawi diabetics and 10 healthy subjects. Capillary glycaemia was measured immediately before the meal and then at 30 min intervals for 3 h. In the diabetic subjects the postprandial glucose response peaked earlier with MOR20 compared to CNT and with lower increments at 90, 120, and 150 min. The mean glycemic meal response with MOR20 was lower than with CNT. The healthy subjects showed no differences. Thus, MO leaf powder could be a hypoglycemic herbal drug. However, given the poor taste acceptability of the 20 g MO meal, lower doses should be evaluated. Moreover, the hypoglycemic effects of MO leaf powder should also be demonstrated by trials evaluating its long-term effects on glycaemia.

## 1. Introduction

*Moringa oleifera* (MO) is a multi-purpose plant native to the Indian subcontinent that, owing to its ability to grow in both humid and hot dry lands and survive in less fertile soils chronically affected by drought, it has become naturalized in tropical and subtropical areas around the world [1,2]. The leaves of this plant are consumed raw or as a powder by many African and Asian populations [3], as they are rich in protein and vitamins, including vitamin A precursors like beta-carotene, minerals, and bioactive compounds [4]. Such chemical-nutritional characteristics make the leaves good candidates for combining with local foods to improve the diet of people living in developing countries, thus reducing the risk of malnutrition. However, it must be said that clinical trials carried out on humans to test the effectiveness of MO in the treatment of malnutrition are limited, and the results are conflicting [5,6,7]. This may depend, at least in part, on the different MO leaf powder doses used in the studies, these being 14–30 g per day for 1–6 months. Indeed, supplementation with too low a dose may not be sufficient to achieve the desired effect, while supplementation with too high a dose, although giving the desired effect, can be unpleasant for the patient, such that it cannot be used outside the research context. In fact, the leaves have a bitter taste that can make food preparations little appreciated. Recent studies have shown that the acceptability of Moringa-based foods decreases as the Moringa amounts increase [8]. Therefore, the identification of the MO leaf powder dose is a critical point to consider before starting a study.

In addition to human nutrition, MO leaves are also used in the traditional medicine of many developing countries as a medical herb to alleviate numerous ailments and treat various diseases, including hyperglycemia and diabetes [1,3]. Several studies in non-diabetic and diabetic animal models have shown that MO leaf, as well as its extracts, could decrease the plasma glucose level and improve glucose tolerance [9,10,11,12,13,14,15]. This hypoglycemic effect has been principally associated to the presence, in the leaves, of fiber and numerous secondary metabolites, such as glucosinolates, isothiocyanates, flavonoids, and phenolic acids, some of which have an amylase inhibition activity [16]. This would reduce the velocity of the starch intestinal digestion and of the glucose intestinal absorption, reducing the postprandial glycemic peak, and thus reducing, presumably, the risk of developing diabetes and improving the management of glycaemia in diabetic subjects. In our experience, adding *Phaseolus vulgaris* extract to starchy meals, this mechanism has proved effective in healthy subjects [17]; on the other hand, the same mechanism is currently used with the administration of acarbose in diabetic subjects. However, studies demonstrating the hypoglycemic effects of MO leaves in humans are, unfortunately, very limited and inconclusive [7,18].

Given the agronomic characteristics of the MO plant, as well as the nutritional composition of MO leaves, refugees from Western Sahara have been cultivating MO trees for some years at the Saharawi refugee camps located in Southwestern Algeria, where they have been living since 1975. Indeed, the Saharawi people are totally dependent on food rations and nutritional supplements provided by the international community for their survival. These rations, distributed monthly, include cereals, pulses, sugar, oil, and blended food [19]. The consumption of fresh fruit and vegetables is, instead, very limited. Owing to this dietary pattern, characterized by a high intake of carbohydrates, especially sugars and complex carbohydrates with a high glycemic index like refined rice, white bread, and pasta, diabetes is the most prevalent health problem in the Saharawi population [19], followed by obesity and stunting in children [20], the latter caused by low intake and quality of proteins. In this context, the use of MO leaves in combination with local food preparations could be useful to increase the intake of nutrients generally lacking in the diet of these individuals and for reducing the glycemic index of meals, presumably reducing the risk of developing diabetes and to improve the management of blood glucose in diabetic subjects.

Thus the aims of this study were to (1) provide a nutritional-chemical characterization of MO leaf powder, (2) evaluate its ability to inhibit the activity of α-amylase, (3) evaluate the sensory acceptability of a local food preparation with added MO leaf powder, and (4) evaluate the effect of the MO leaf powder, produced locally at Saharawi refugee camps and added to a local food preparation, on the postprandial glucose response in Saharawi diabetic and healthy subjects. 

## 2. Materials and Methods 

### 2.1. Study Design

The present study consisted of four different steps.

- Step 1—Chemical assessment to nutritionally characterize the MO leaf powder produced at Saharawi refugee camps.
- Step 2—Enzyme assay to evaluate the ability of MO leaf powder extract to inhibit α-amylase activity.
- Step 3—Acceptability test to evaluate the sensory characteristics and the overall acceptability of a traditional meal added with 20 g of MO leaf powder.
- Step 4—Glycemic response test to evaluate how a traditional meal with an added 20 g of MO leaf powder affects postprandial glucose response in diabetic and non-diabetic subjects.

The chemical assessment of MO leaf powder and the enzyme assay were conducted at the Department of Food, Environmental and Nutritional Sciences (DeFENS), University of Milan, whilst the in vivo tests were conducted at the dispensary of Tifariti at Smara camp (Saharawi refugee camps, Tindouf province, South-Western Algeria). 

### 2.2. Sample

MO leaves were collected in November 2017 from Moringa trees experimentally cultivated at the Centro Experimental de Formación Agrícola/Experimental Center of Agricultural Formation (CEFA), an oasis created in 2009 next to the Rabouni camp (Tindouf, South-Western Algeria). The underground water resources were used to cultivate vegetables and trees for livestock and human nutrition. The leaves were dried through a shade-dried process at room temperature, and ground to a fine powder with an electric grinder. 

### 2.3. Subjects

The subjects were Saharawi volunteers, people living at Smara camp (Tindouf province, South-Western Algeria), recruited in January 2018 from among non-diabetic and diabetic subjects. The recruitment was done by the Ministerio de Salud Pública (Ministry of Public Health) of Sahrawi Arab Democratic Republic. The healthy subjects were selected among subjects living near the dispensary of Tifariti at Smara camp through advertisements in the local community. They had to be in apparent good health and of normal weight, and were not undergoing any medications for chronic use. The diabetic patients were selected among patients followed by the dispensary of Tifariti at Smara camp. The subjects eligible for the study had to be diagnosed type 2 diabetes for at least 1 year, had to be free of advanced retinopathy, terminal renal failure, active diabetic ulcers, amputations, and heart failure, and not undergoing insulin therapy, being treated only with oral hypoglycemic agents. The present study was conducted according to the guidelines laid down in the Declaration of Helsinki. The Ministerio de Salud Pública (Ministry of Public Health) of Sahrawi Arab Democratic Republic approved the study procedures (approval n. 88/2017), and each subject gave written informed consent to participate in the study. 

### 2.4. Meal Preparation

A traditional meal consisted of 80 g of white rice and 160 g of camel meat stew (raw weights). The rice, camel meat, and all the other ingredients were bought at the local dealers. Each portion of rice was cooked in 1 L of boiling water with 5 g of salt for 15 min. Each portion of the camel meat for the stew was first sautéed with onions, carrots, garlic, tomatoes in sunflower oil for 15 min, water was then added and the pot left to stew for 2 h. The meals were prepared in two portions for each subject, one portion was supplemented with 20 g of MO leaf powder (MOR20) and the other one was administrated without MO leaf powder (control meal, CNT). We selected the dose of 20 g because it is within the range reported in literature, and according to our previous informal experience, this dose was acceptable from a sensorial point of view and easily consumable in case of a chronic use.

### 2.5. Experimental Protocol

#### 2.5.1. Chemical Assessment

200 g of leaf powder were vacuum-packed and sent to the DeFENS laboratories, where the proximate analysis and the determination of any secondary metabolites were performed. The residual moisture content was determined by drying the samples at 105 °C for 6 h. The ash content was determined by incineration of the samples at 550 °C for 6 h. The minerals content was determined by inductively coupled plasma mass spectrometry (ICP-MS). The Kjeldahl and Soxlet methods were respectively used to determine protein and lipid content [21]. Prosky’s method was used to determine the soluble and insoluble fiber content [22]. Sugar content was determined by means of anion exchange high performance liquid chromatography (HPLC) with pulsed amperometric detection [23], as previously described in Reference [4]. The total glucosinolate, phenol, and saponines content was determined by UV-Vis spectrophotometry.

The spectrophotometric estimation of the total gluconinolates was done according to Mawlong et al. [24] in aqueous methanol extracts. Portions of 1 g of leaf powder were extracted with methanol 55% (1/20 g drug/mL solvent) at 80 °C for 15 min. The extraction was repeated twice. The extracts were prepared in triplicate. A 100 μL volume of each extract was added with 0.3 mL double-distilled water and 3 mL of 2 mM sodium tetrachloropalladate (58.8 mg sodium tetrachloropalladate + 170 μL concentrated HCl + 100 mL double distilled water). After incubation at room temperature for 1 h, the sample absorbance was measured at 425 nm. The calibration curve was determined with sinigrin as standard (Sigma-Aldrich, Milano, Italy), with concentrations ranging from 0.50 to 2.00 mM. The total glucosinolate content was expressed as mg of sinigrin equivalent (SE)/g dry weight material (DW).

The total phenol quantification was performed on the methanol extract of dry leaves [25], and the total phenol content determined using Folin-Ciocalteu reagent and gallic acid used as standard. A sample of 200 μL of extract was introduced into screw cap test tubes and added with 7.8 mL of water, 0.5 mL of Folin-Ciocalteu reagent, and after 3 min, 1.0 mL of Na_2_CO_3_ (20% solution). The tubes were vortexed, then set in the dark at room temperature for 2 h. The calibration curve was determined with gallic acid solutions using concentrations ranging from 100 to 1600 μM. Absorption at 726 nm was measured and the total phenol content expressed as mg of gallic acid equivalents (GAE) per g of DW. The analysis was done in triplicate.

Total saponin content was measured according to Lozano et al. [26]. A saponin methanol:water (80%) extract (1.0 mL) was placed in a tube with 1.0 mL of the Lieberman-Burchard (LB) reagent (acetic anhydride 16.7% in concentrated sulfuric acid) for color development, vortexed vigorously (30 s), and then left at room temperature for 30 min. Absorbance at 528 nm was measured. The total saponin content was obtained by comparison with a standard curve of oleanolic acid (17 μg/mL to 350 μg/mL), and the results expressed as mg of oleanolic acid equivalent/g DW. The analysis was done in triplicate.

#### 2.5.2. Enzyme Assay

Five grams of MO leaf powder were extracted twice (48 h each time), using 12 volumes of methanol at 65 °C in the presence of a condenser and under constant magnetic stirring. After filtration, the methanol was removed by evaporation under vacuum and stored at −30 °C. Appropriate amounts of dried extract were freshly diluted in methanol for the enzyme assay.

The α-amylase activity measurement was carried out by the colorimetric determination of starch reducing-ends formed during the enzyme reaction, using the DNS reagent in a method modified from Bernfeld [27]. The assay mixture was prepared by sequentially adding 20 µL of methanol-diluted MO extract (sample), or methanol-diluted acarbose (A8980, Sigma-Aldrich, Milano, Italy), or methanol (inhibition-negative control), 80 µL of 2 U/mL porcine pancreatic α-amylase (A3176, Sigma-Aldrich, Milano, Italy) stock in 0.2 M NaH_2_PO_4_, 10 mM NaCl, pH 6.9, and then pre-equilibration 15 min at 37 °C in a water bath, 300 µL of 5 mg/mL soluble potato starch (freshly prepared in boiling water and stored briefly at 55 °C in the water bath till use). The enzyme reaction was stopped after 30 min by the addition of 200 µL of DNS reagent, and was incubated at 96 °C for 15 min. After dilution with 1 mL water, the absorbance at 540 nm was determined. The DNS reagent (38 mM 3,5-dinitrosalicylic acid, 0.85 M sodium potassium tartrate, 0.08 M NaOH) was prepared combining sodium potassium tartrate dissolved in 8 mL of 2 M NaOH and 3,5-dinitrosalicylic acid dissolved in 20 mL of water, and adjusting to the final volume (50 mL) with water. In the enzyme-negative controls, the enzyme was replaced by 0.2 M NaH_2_PO_4_, 10 mM NaCl, pH 6.9. The elaboration of the percent inhibition values was carried out on the net absorbance values subtracted from those of the corresponding enzyme-negative controls. The concentration of the extract required to inhibit 50% of the α-amylase activity under the assay conditions was defined as the IC_50_ value. The IC_50_ values were extrapolated fitting a four-parameter logistic function on plotted dose-response data.

#### 2.5.3. Acceptability Test

The day of the test, the healthy and diabetic subjects arrived at the dispensary of Tifariti at Smara camp at 09:30 AM, and were seated in a comfortable room. Then, one by one, they were accommodated in an adjacent room where several testing cabins were set up. Each subject was allocated in a different cabin and was asked to consume the 15 g of MOR20 and CNT. The samples were presented in a computer generated randomized order and the subjects was asked to test the samples following this sequence. After consuming the first sample, and rating it, the subjects sipped water before going on to taste the second sample. For the sample rating, the subjects were asked to evaluate the sample’s color, taste, texture, and overall acceptability using a 9-cm linear scale, with anchors of “dislike extremely” on the left and “like extremely” on the right. Participants and investigators were not blinded during the test because the addition of MO leaf powder made the testing meal green, and therefore, easily recognizable.

#### 2.5.4. Glycemic Response Test

On two different days and in computer generated random order, each subject received two identical looking meals differing only in the addition of a definite amount of 20 g of MO. On the evening before each experiment, the subjects were asked to consume the last meal before the experiment by 9 p.m. The evening meal had to be composed of the usual foods in the usual quantities, according to each subject’s dietary habits. After 9 p.m., only water was allowed. The diabetic subjects were instructed to take the last hypoglycemic medications before midnight of the day prior to the study. At 9:30 a.m. of the subsequent day, the subjects arrived at the dispensary of Tifariti at Smara camp and were seated in a comfortable room where the fasting capillary whole blood was obtained by finger prick (A. Menarini Diagnostics s.r.l, Firenze, Italy) and the glucose concentration was measured by means of a glucometer GlucoMen LX 2 (A. Menarini Diagnostics s.r.l, Firenze, Italy), with glucose-oxidase-based test strips (A. Menarini Diagnostics s.r.l, Firenze, Italy). Immediately after this, each subject received the test meal, and at 30, 60, 90, 120, 150, and 180 min from the beginning of the meal, the capillary blood glucose level was measured as described above. During the meal consumption, all the subjects were monitored by the nurse working at the dispensary to ensure that they completed the meal within 20 min. Moreover, in this case, the participants were not blinded, but the physicians who performed the blood samplings were not informed about which meal the subjects consumed.

### 2.6. Statistical Analysis

Continuous variables were checked for normality. Data regarding the general characteristics of the recruited subjects (age and BMI) were reported as mean and standard deviation, whilst the data regarding the chemical and sensory measurements and the postprandial glucose response were presented as mean and standard error. MOR20 and CNT were compared in terms of sensory characteristics and postprandial glucose responses using a paired *T*-Test. The postprandial glucose response was assessed using the capillary glycaemia expresses as concentration and as change from the baseline at individual time points, the maximal glucose concentration, the time of maximal peak, and the mean glucose response. The latter refers to the average of the glycemic values at all time-points of OGTT for a given individual. A *p*-value < 0.05 was considered as statistically significant. Statistical analysis was performed using STATA version 12.0 (StataCorp, College Station, TX, USA).

## 3. Results

### 3.1. Chemical Assessment

The MO leaf powder had a residual moisture of 5.6 g/100 g, and its nutritional composition is shown in Table 1.

### 3.2. Enzyme Assay

**Figure 1:**
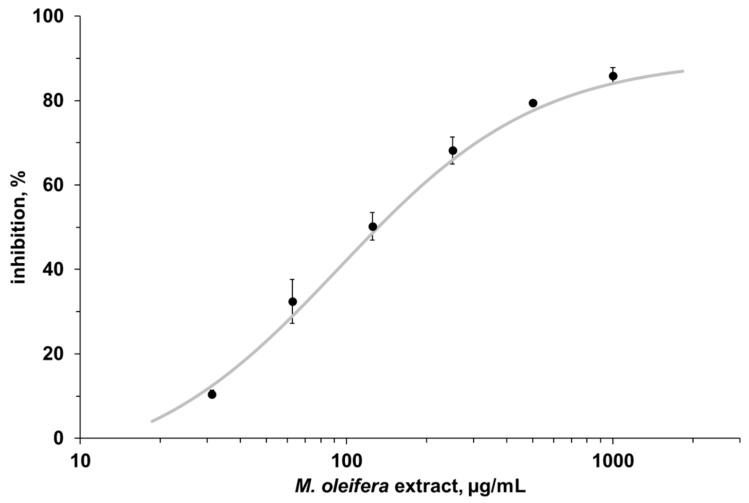
Representative inhibition profile of α-amylase activity in the presence of *Moringa oleifera* extract.

The activity of α-amylase was assessed in the presence of MO leaf extract (250 µg/mL), and it was found that there was a 68.2 ± 3.2% decrease, compared to the α-amylase activity measured in the absence of the MO extract. Indeed, the inhibition profile (Figure 1) of the measured α-amylase activity, carried out in the presence of different MO extract concentrations, ranging from 30 to 1000 µg/mL, led to the determination of an IC_50_ value of 220 ± 17 µg/mL. The minimum MO extract concentration to cause clear inhibition of the α-amylase activity was 120 ± 5 µg/mL. Using the same assay, acarbose, a synthetic drug proposed for managing postprandial blood glucose levels, gave an IC_50_ value of 13.2 ± 1.4 µg/mL (20.5 ± 2.1 µM).

### 3.3. Acceptability and Glycemic Response Tests

**Figure 2:**
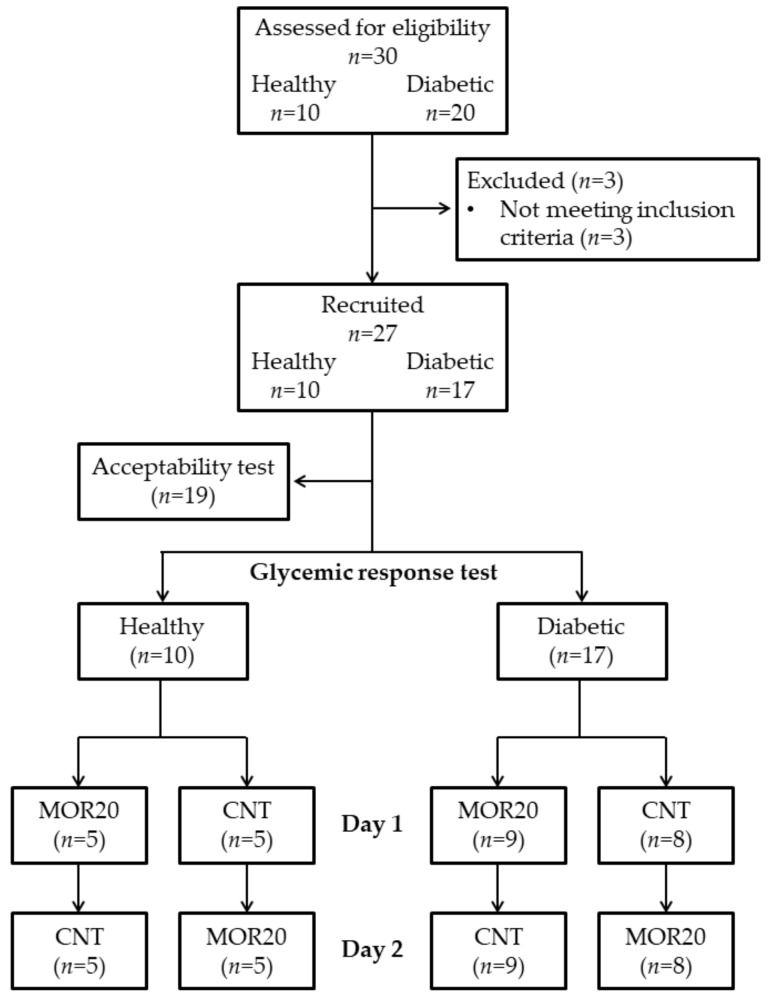
Flowchart of the study plan; MOR20: Meal supplemented with 20 g of *Moringa oleifera* leaf powder; CNT: Control meal.

The flowchart of the study plan is reported in Figure 2.

Assessed for eligibility were 30 people (10 healthy and 20 diabetic subjects). Of these, 3 diabetic subjects were excluded because they did not meet the inclusion criteria. Therefore, 27 subjects were recruited for the study. The healthy subjects (*n* = 10) were 4 males and 6 females, aged 42 ± 11 years. The diabetic subjects (*n* = 17) were 8 males and 9 females, aged 62 ± 9 years, and on average, mildly overweight (BMI: 25.2 ± 4.3 kg/m^2^). Their diabetes was diagnosed 10 ± 6 years earlier and they were currently being treated with Metformin and Glibenclamide, alone or in combination, with the exception of one subject who was not undergoing any treatment. Four were concomitantly being treated for hypertension but none for dyslipidemia.

Of the 27 subjects recruited for the study, a subgroup of 19 subjects (9 diabetics and 10 healthy, 8 males and 11 females, age: 50 ± 13 years) participated in the acceptability test, whilst all the recruited subjects participated in the glycemic response test.

#### 3.3.1. Acceptability Test

The subjects who participated in the acceptability test evaluated the test meal with and without the addition of 20 g of MO leaf powder regarding color, taste, texture, and overall acceptability. Table 2 shows the results, and it can be seen that the ratings of color and taste, but not texture, were generally reduced when the leaf powder was added. Moreover, the overall acceptability was reduced in a non-statistically significant manner (*p* = 0.055).

#### 3.3.2. Glycemic Response Test

**Figure 3:**
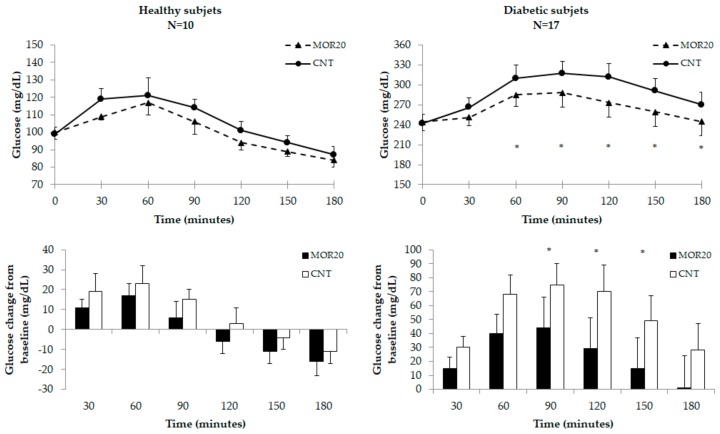
Kinetics and changes from the baseline of capillary blood glucose after administration of a meal with added 20 g of MO leaf powder (MOR20) and a control meal (CNT) in healthy and diabetic subjects. Values are reported in the graphs are means and standard errors. * *p* < 0.05.

Figure 3 shows the kinetics and changes from the baseline of capillary blood glucose after administration of MOR20 and CNT in healthy and diabetic subjects. 

In healthy subjects, the baseline blood glucose was similar on both occasions. Postprandial blood glucose peaked at similar times and concentrations after MOR20 and CNT (57 ± 7 min vs. 51 ± 8 min, *p* = 0.619; 121 ± 5 mg/dL vs. 135 ± 7 mg/dL, *p* = 0.067), and no significant differences in either absolute and incremental glucose concentrations were observed at any time point. The mean meal glycemic response with MOR20 (100 ± 3 mg/dL) did not differ from that with CNT (106 ± 3 mg/dL, *p* = 0.145). 

In diabetic subjects, baseline blood glucose was similar during the two experimentations. The postprandial glucose response peaked earlier and at lower concentrations with MOR20 than with CNT (67 ± 8 min vs. 90 ± 10 min, *p* = 0.018; 315 ± 15 mg/dL vs 340 ± 18 mg/dL; *p* = 0.003). Starting from 60 min from the beginning of the meal, blood glucose concentrations were always lower with MOR20 compared to CNT. We observed a lower increment from basal of blood glucose with MOR20 compared to CNT at 90 min (+44 ± 22 mg/dL vs. +75 ± 15 mg/dL, *p* = 0.036), 120 min (+29 ± 22 mg/dL vs. +70 ± 19 mg/dL, *p* = 0.003) and 150 min (+15 ± 22 mg/dL vs. +49 ± 18 mg/dL, *p* = 0.014). The mean glycemic meal response was lower with MOR20 (268 ± 18 mg/dL) than that obtained with CNT (296 ± 17 mg/dL, *p* < 0.001).

## 4. Discussion

In this study, we provide the first evidence of a hypoglycemic effect of MO leaf powder, produced locally in Saharawi refugee camps, added to a local food preparation, on the postprandial glucose response in Saharawi diabetic and healthy subjects. To achieve this goal, we initially evaluated the nutritional-chemical composition of the MO leaf powder, its α-amylase-inhibition activity, and its sensory acceptability in local food preparations. 

As expected, proteins were the predominant nutrient along with fiber, and represent, approximately, 31% and 33% of the leaf powder. The leaf powder was also found to be rich in calcium, potassium, and iron, and as expected, their content was greater than that found in the leaves of MO trees cultivated in other countries of the world [4,5]. This is presumably due to the adverse environmental conditions to which the plant is subject to at Saharawi refugee camps, such as the low water availability, soil salinity, and scarcity of rain, which led the plant to increase the presence of minerals to deal with the increased osmotic stresses [28,29]. Total polyphenol content was within the range reported in the literature for different samples of Moringa leaves, which varies a lot depending on geographic origin, age, wild or domesticated plant status, and method of determination. The value is slightly lower than that already reported by some of us, in Moringa from Saharawi [4] (35.5 mg GAE/g DW), and by other authors (36–45 mg GAE/g DW) in tender and mature leaves, respectively [30]. The saponins content in our sample was 16.92 OAE/g DW. The dried leaves had a total content of glucosinolates of 21.22 mg SE/g DW, a low value like that was found by high performance liquid chromatography-mass spectrometry (LC-MS) [31] in several samples of young and old leaves coming from cultivated and wild samples of Moringa in different geographic areas, ranging from 41 and 116 mg/g. With the same HPLC method that we used, and considering the sum of the two main peaks of principal glucosinolates, Chodur at al. [32] obtained two extremely different glucosinolates values for samples from Kenya (1.16 mg/g) and Mexico (109 mg/g). 

The result of the in vitro inhibition test displayed that the extract reduced the activity of α-amylase, a key enzyme responsible for the digestion of dietary carbohydrates to glucose, suggesting that MO leaf powder decreased the postprandial glucose level by reducing the velocity of the amylase-mediated starch hydrolysis, and, that of glucose intestinal absorption. The results of the enzymatic assay confirmed what was already obtained in a previous study, where the aqueous extract of MO leaves inhibited the activity of α-amylase. However, in our study, the extract concentration needed to inhibit the 50% of the α-amylase activity was 4-fold higher (220 µg/mL vs. 52.5 µg/mL) [13], and this difference could depend on a different concentration of bioactive compounds.

Numerous articles and reviews have suggested the use of MO leaves in combination with other local foods to enrich, in nutrients and bioactive compounds, the diet of people living in developing countries. However, given its bitter taste, it is important to evaluate the sensory acceptability of a traditional meal supplemented with MO leaf powder. The dislike of MO leaf flavor in food would be the first cause of failure of any clinical trial. Nevertheless, studies that have assessed the sensory acceptability of MO leaves in food are very limited. Our test shows that adding 20 g of MO leaf powder, corresponding to 8% of the meal’s weight, resulted in a significant reduction in meal acceptability with regard to color and taste, but not texture, in agreement with recent findings showing a decrement in the sensory characteristics acceptability of Moringa-added foods [8]. However, we found that the meal added with MO leaf powder was accepted by the tasting panel. Recently, some of us found that a daily dose of 14 g of MO leaf powder split in the two main meals was well tolerated by a group of Zambian malnourished girls aged 2–20 years [5]. In another study, the addition of 35 g, corresponding to 15% of the meal’s weight, of MO leaf powder to a meal consisting of a cereal-legume blend was generally well accepted by a group of Ghanaian infants and their caregivers [33]. Therefore, our result agrees with findings reported in the literature. However, it is very likely that larger amounts of MO leaf powder result in an excessively unpleasant taste, due to the bitterness of the leaves.

Finally, we evaluated the effect of a MO leaf powder supplemented meal on the postprandial glycemic response in diabetic and healthy subjects. We found that the leaf supplementation determined a lower increment of the postprandial blood glucose in diabetic subjects, 90 min from the beginning of the meal. Overall, diabetic subjects had a mean change from the baseline of postprandial glucose concentration of ≈30 mg/dL lower when the meal was supplemented with MO leaf powder, compared to the control meal. Contrarily, no effect was found in non-diabetic subjects. 

Previous in vivo studies have shown the ability of MO to reduce postprandial glucose in mice, either by treating them with a single dose of MO administered concomitantly to the meal [9], or by pre-treating the mice with MO over a given period of time [11]. In humans, in a single dose study with six type 2 diabetic subjects, the consumption of 50 g of MO cooked leaves together with a standard meal significantly decreased blood glucose levels by 21% one hour from the beginning of the meal, whereas at 2 h the difference was no longer significant [34]. In addition, no alteration in insulin secretion was observed. In our study, adding 20 g of MO leaf powder to the meal resulted in a reduced postprandial glucose response that was maintained for up to 3 h from the beginning of the meal. Thus, this different effect of MO on glycemic response could be related to the amount of leaf provided during the study and the way of administration. Indeed, considering a moisture content of 76–78%, 50 g of fresh leaf corresponds to 11–12 g of dry leaf, a smaller amount than that used in our study. Moreover, cooking causes a loss of nutrients and bioactive compounds, which may be preserved by adding the leaf powder to the meal after cooking. 

The hypoglycemic effect of MO leaf powder has been postulated to be associated with the presence, in the leaf, of fiber and numerous secondary metabolites. Indeed, the high fiber content of MO leaf determines a slowing of glucose intestinal uptake and of gastric emptying time. Instead secondary metabolites, like flavonoids and phenolic acids, have been involved in carbohydrate metabolism as they inhibited the α-glucosidase and α-amylase [35,36]. Rutin inhibited α-glycosidase activity in vitro by directly binding to the enzyme through hydrophobic bonding [37]. In addition, kaempferol inhibited the α-glycosidase activity in vitro by binding to the enzyme through hydrogen bonds and van der Waals forces, and this binding resulted in a conformational alteration of α-glucosidase [38]. Quercetin displayed more inhibitory activities through the inhibition of both maltase and sucrase activities in vitro and in vivo [39]. Moreover, quercetin inhibited GLUT2-mediated uptake in vitro and reduced postprandial blood glucose levels in diabetic mice when it was orally administered with glucose compared to glucose only administration [40]. Similarly, also some phenolic acids, like chlorogenic, ferulic, caffeic, and tannic acids, could interact with absorption of glucose from intestine via inhibition of sodium-dependent SGLT1-mediated glucose transporter [41]. The inhibition of amylolytic enzymes slows the digestion of carbohydrates and reduces the rate of glucose increment in the blood stream, addressing a possible explanation to the postprandial glucose response results. The isothiocyanates are other secondary metabolites present in the MO leaves. These compounds derive by the myrosinase-mediated hydrolysis of glucosinolates and have shown anti-diabetic properties, as they have been demonstrated to inhibit the liver gluconeogenesis [42,43].

Given that MO leaf powder is able to reduce postprandial glycemic response, it is plausible to think that the constant use of MO leaf powder can improve the management of glycaemia in diabetic subjects. To date, the effects of a constant consumption of MO leaf powder on blood glucose in humans have been little studied. If demonstrated, such activity would be peculiar to populations that, due to environmental, political, and economic reasons, have reduced access to Western drug therapies. For instance, the Saharawi people are a traditionally nomadic population that for over 40 years has been living in a protracted refugee setting, where the population has not experienced economic development and is dependent on food assistance for survival. A typical food assistance basket for this population includes starchy foods (refined grain cereals, pulses, and blended foods) and sugar, with low quantities, if any, of fresh or dried vegetables and fruit. This unbalanced low-diversity diet is, presumably, a main cause of the nutritional deficiencies, especially of vitamins and minerals, recorded in children [44], as well as of the high prevalence of stunting and general and central obesity observed in Saharawi children and women, respectively [20]. Obesity and the high consumption of refined carbohydrates and sugars are the main causes of the development of type 2 diabetes. Among the Saharawi population diabetes is a prevailing health problem that is generally treated either with diet or diet plus hypoglycemic agents. In this context, MO leaves could be used as a food supplement to improve diet quality and the management of glycaemia in diabetic subjects. For this reason, we selected the Saharawi people as the target population in which to evaluate the sensory acceptability and the effects on blood glucose of a meal supplemented with locally produced MO leaf powder. This represents the first strength of our study. Secondly, for the first time we evaluated the hypoglycemic effects of MO leaf powder in humans on a consistent number of diabetic and non-diabetic subjects. However, our study is not free of limitations. For one thing, we measured capillary, not venous, blood glucose. Although comparison studies have found small but significant differences in the glycemic values obtained from the two methods [45,46], the measurement of capillary blood glucose is a simple and rapid method, and has been selected for its unique convenience due to the reduced availability of laboratory equipment and reagents present on site. For the same reason, insulin response to the meal could not be measured, and this represents the second limitation of our study. Finally, we did not record what the subjects really consumed the evening before the experiment. However, it should be considered that the food available at the refugee camps is that of the food relief packages, therefore, we can assume that the participants consumed roughly the same foods.

## 5. Conclusions

The MO leaf powder was rich in nutrients whose intakes in subjects living in developing countries generally do not meet the recommended levels, and therefore, MO use in typical meals could improve diet quality, and consequently, the nutritional status, body composition, and risk of chronic disease in such populations. However, clinical trials on this topic are still limited and inconclusive, and further studies are very much required. For the local populations the addition of a contained MO leaf powder dose to local foods seems to be tolerable. However, larger doses could make the food preparations unpleasant due to the bitter taste of the leaves. Therefore, it is advisable to perform acceptability tests before starting any trial, to identify the maximum tolerated dose. The MO leaf powder seems to be able to reduce the postprandial glucose response in diabetic subjects, and this activity is potentially related, at least in part, to the inhibition of α-amylase activity. This makes it a prospective herbal drug to be used among populations in developing countries, in combination with the Western therapies currently available. However, before being recognized as an herbal drug, the hypoglycemic effects of MO leaf powder must to be demonstrated by clinical trials evaluating the powder’s long-term effects on blood glucose. 

## Figures and Tables

**Table 1:** Nutritional-chemical composition of *Moringa oleifera* (MO) leaf powder.

Nutrients		
Proteins	g/100 g	30.6 ± 0.8
Lipids	g/100 g	5.6 ± 0.3
Total fiber	g/100 g	32.8 ± 0.2
Soluble fiber	g/100 g	5.7 ± 0.1
Insoluble fiber	g/100 g	27.1 ± 0.2
Starch (estimated by difference)	g/100 g	11.3
Sugars	g/100 g	4.6 ± 0.1
Ash	g/100 g	15.1 ± 0.3
Sodium	mg/100 g	502 ± 5
Potassium	mg/100 g	1492 ± 13
Calcium	mg/100 g	2997 ± 27
Iron	mg/100 g	30.2 ± 0.3
Total polyphenols	mg GAE/g	23.91 ± 0.2
Total glucosinolates	mg SE/g	21.22 ± 3.7
Total saponins	mg OAE/g	16.92 ± 0.6

Values are means and standard errors expressed as grams of dry weight material; GAE: Gallic Acid Equivalents; SE: Sinigrin Equivalents; OAE: Oleanoic Acid Equivalents.

**Table 2:** Sensory characteristics and overall acceptability of the meals supplemented and not supplemented, with MO leaf powder.

Sensory Characteristics	MOR20	CNT	*p*-Value
Color	5.0 ± 0.5	6.6 ± 0.5	0.003
Taste	5.4 ± 0.4	6.4 ± 0.4	0.024
Texture	5.8 ± 0.6	6.8 ± 0.6	0.064
Acceptability	5.2 ± 0.5	6.4 ± 0.5	0.055

Values are mean and standard errors. Values are scores obtained using a 9-cm linear scale, with anchors of “dislike extremely” on the left and “like extremely” on the right. Means were compared using the paired *T*-Test.
